# A rapid method for the generation of uniform acellular bone explants: a technical note

**DOI:** 10.1186/1749-799X-5-32

**Published:** 2010-05-10

**Authors:** Katharina Jähn, Volker Braunstein, Pamela I Furlong, Angharad E Simpson, R Geoff Richards, Martin J Stoddart

**Affiliations:** 1 AO Research Institute, Clavadelerstrasse 8, 7270 Davos, Switzerland; 2 Cardiff University, School of Biosciences, Cardiff University, Cardiff, (UK), CF103AX; 3 Chirurgische Klinik und Poliklinik Innenstadt, Nußbaumstr. 20, 80336, München, Germany

## Abstract

**Background:**

Bone graft studies lack standardized controls. We aim to present a quick and reliable method for the intra-operative generation of acellular bone explants.

**Methods:**

Therefore, ovine cancellous bone explants from the iliac crest were prepared and used to test several methods for the induction of cell death. Over night heat inactivation was used as positive treatment control, methods to be investigated included UV light, or X- ray exposure, incubation in a hypotonic solution (salt-free water) and a short cycle of repeated freezing and thawing.

**Results:**

Viability of treated and 2 days cultured bone explants was investigated by lactate dehydrogenase assay. Non-treated cultured control explants maintained around 50% osteocyte viability, while osteocyte survival after the positive treatment control was abolished. The most dramatic loss in cell viability, together with a low standard deviation, was a repeated cycle of freezing and thawing.

**Conclusions:**

To summarize, we present a freeze-thaw method for the creation of acellular bone explants, which is easy to perform, not time-consuming and provides consistent results.

## Background

Large bone defects remain a clinical challenge and the development of novel therapies requires adequate controls. The use of acellular bone explants within bone graft studies is of crucial importance [1]. Results of transplanted bone material can not be interpreted correctly without the presence of a bone matrix control which does not contain viable cells. It is well known that the success of bone grafts is determined by three factors - osteoinduction, osteoconductivity and osteogenesis [1]. The concept of osteoinduction involves mitogenesis of undifferentiated host mesenchymal stem cells towards the formation of osteoprogenitors by i.e. molecules of the TGFβ family which are stored in huge amounts within bone matrix [2]. Osteoconductivity is achieved when the implanted bone is used by the host cells as scaffold for the formation of new bone. An acellular bone explant control aids to evaluate the effects of active bone cells (osteogenesis) versus the effect of the bone matrix alone (osteoinduction and osteoconductivity) during bone transplantation studies. Unfortunately, a reliable control with a uniformly diminished cell survival is not standardized yet.

Acellular bone grafts are often prepared using γ-irradiation or ethylene oxide sterilization of allogenic bone explant material [3]. Yet, both methods have been shown to reduce the mechanical properties of bone matrix and negatively affect osteogenesis by the host cells due to either residual ethylene oxide or radiation effects. The use of demineralized bone grafts, available from tissue banks, is very popular due to the ease of purchasing. The acid extracted (demineralized) bone allograft is of acellular type. However, the demineralization process is impractically long, if this method would be intended to be used for the generation of acellular autologous bone material intraoperative.

Therefore, the aim of this study is to present a reliable, quick and easy method for the generation of an acellular bone explant control, which could be performed during transplantation operation on possibly autologous bone. The method must routinely result in a totally acellular sample. We demonstrate that a rapid cycle of freeze-thawing is more effective over the use of radiation exposure (X-ray, or UV-C light) or incubation in salt-free water, and less time consuming as an over night incubation at high temperatures (>50°C) to generate uniformly acellular cancellous bone.

## Methods

### Cancellous Bone Harvest and Treatment

Cancellous bone was obtained from the iliac crest of 3 Swiss Alpine sheep, which were euthanized due to involvement in separate studies (approved by cantonal ethics committee). Cylindrical bone explants were drilled from the iliac crest using a 9.5 mm Synthes drill bit (Ref: 387.661, Synthes, Bettlach, Switzerland). Bone explants were randomly assigned into 6 different treatment groups with 3 explants per group. The first treatment group was exposed to 5 freeze-thaw cycles. Therefore cores were put in a metal beaker and placed into liquid nitrogen for 1 min. After each freezing cycle, explants were thawed at 56°C for 5 min. The second and third treatment groups were exposed to radiation. While explants of one group were treated with 80 kV X-rays for 30 min, group 3 was placed 20 cm from an UV-C light source (Osram HNS, 30 W) for 60 min. The last group was placed in salt-free water (purified with Mili-Q Synthesis) for 1 h treatment. The positive treatment control group was incubated at 56°C for 18 h.

### Culture and Viability Analysis

**Figure 1:**
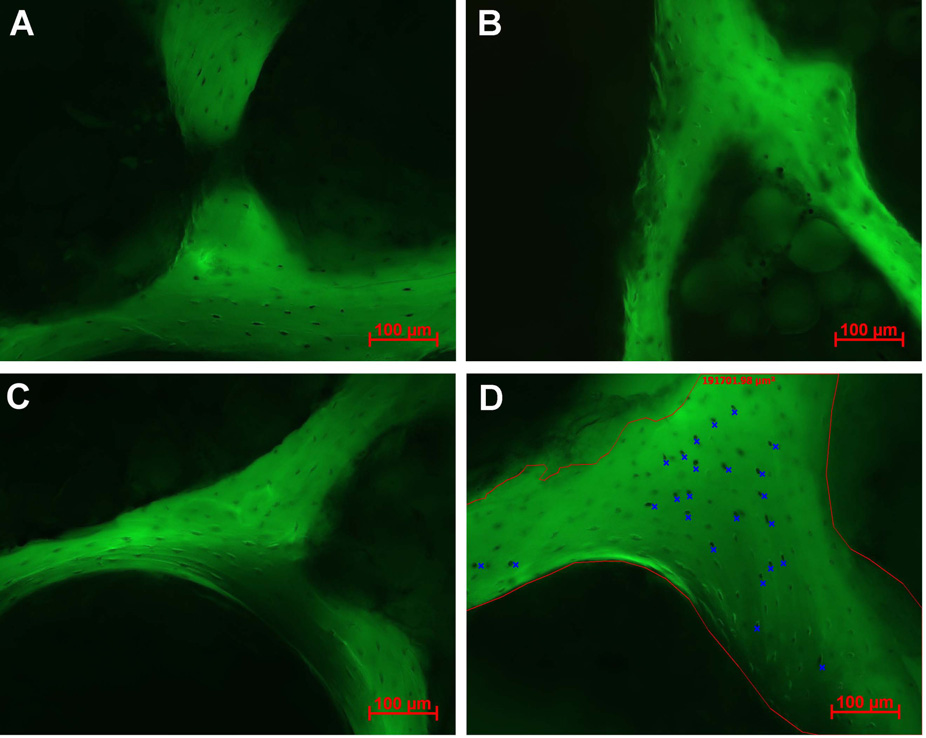
**Four representative micrographs from LDH labeled ovine cancellous bone sections visualized with Axioplan (Fluorescence 515-565 emission filter)**. **A**: Non-treatment control cultured for 2 days with presence of viable osteocytes; **B**: Positive treatment control incubated at 56°C over night prior to 2 days culture; **C**: Freeze-thawing treatment after 2 days culture showing no viable osteocytes; **D**: Representative image to show the method of analysis - bone matrix area is marked in red, viable osteocytes are marked in blue.

All treated bone explants, together with a non-treatment control group were cultured for 2 d in DMEM high glucose (Invitrogen) supplemented with 4 mM sodium hydrogen carbonate (Merck), 10 mM HEPES, 5 mM glycerol-2-phoshate salt hydrate (Sigma), 10 mg/l L-ascorbic acid phosphate magnesium salt n-hydrate (Wako Chemicals), 50,000 U/l Penicillin, 50 mg/l Streptomycin (Invitrogen), and 10% fetal calf serum (FCS; South American origin, biowest). Viability was analyzed using a lactate dehydrogenase (LDH) assay [4], with a day 0 non-culture group to provide baseline viability levels. Harvested explants were cut with a Leica annular saw (Leica AG, Glattbrugg, Switzerland) into 300 μm sections. LDH staining was performed using a 5% Polypep base solution containing 0.75% sodium chloride and 2 mM Glycyl-Glycine buffer (adjust to pH 8). Lactic acid (60 mM), 1.75 mg/ml NAD^+ ^(pH 8), and 3 mg/ml nitroblue tetrazolium (all Sigma) were added freshly to the solution on the day of the assay. Visualization and quantification of viable osteocytes per mm^2 ^bone area was performed as previously described [5]. In brief, fluorescence micrographs (excitation BP450-490 nm, beam splitter FT510 nm, emission BP515-565 nm) were taken from the central regions of the bone explants using a 20× objective. Bone matrix area per micrograph was determined. Due to the achieved depth of field of 4.12-4.52 μm, dark violet stained osteocytes in focus could be accounted as viable osteocytes per bone area [Fig. 1D].

### Statistical Analysis

The Mann-Whitney Rank Sum test and Bonferroni correction was used to compare the different methods, which were analyzed to generate acellular cancellous bone samples. A p-value ≤ 0.05 was considered to be significant.

## Results

Due to the fact that there is so much cell death at the periphery induced during the cutting process, we did not analyze this region to remove any effect of preparation artifact. Macroscopical analysis of LDH viability staining on non-cultured bone explants, showed overall uniform cell viability. Whereas viability of non-treated 2 days cultured bone demonstrated a central area of decreased marrow survival. Macroscopically, all treatment groups, cultured on for 2 days, showed almost no detectable marrow LDH viability staining (data not shown).

Osteocyte survival was determined and quantified at higher magnification. Presence of formazan crystals within osteocytes demonstrating cellular LDH activity and therefore cell viability was demonstrated in non-cultured and non-treated bone explants. The positive 56°C heat incubation treatment group, as well as other treatment groups, presented no or decreased osteocyte LDH activity [for representative images see Fig. 1].

**Figure 2:**
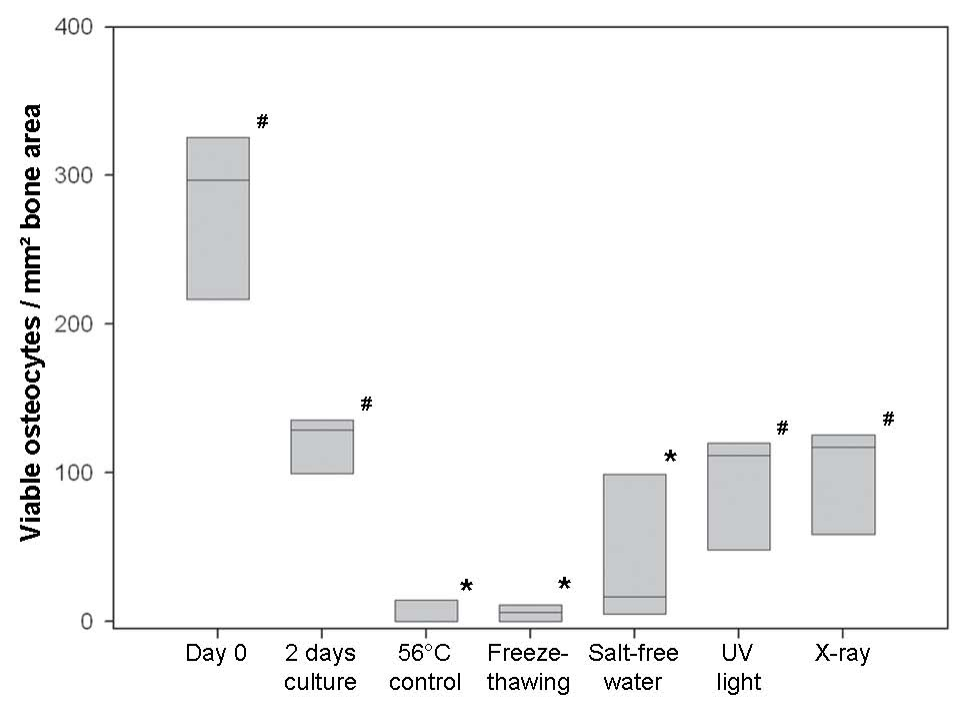
**Graph shows boxplots of the datasets for viable osteocytes per bone matrix area under the different treatment conditions**. Osteocyte survival was greatly diminished during 56°C over night heat incubation, freeze-thawing and salt-free water treatment compared to non-treated 2 days culture control (* p ≤ 0.05). Moreover, UV light and X-ray exposure were significantly different to the positive treatment control of 56°C incubation (# p ≤ 0.05). Boxplots show the median line, the 25% and the 75% quartiles which define the box.

During static culture of 2 days the average cell viability of bone explants dropped over 50% from 279 (+/- 58) to 119 (+/- 25) viable osteocytes per bone matrix area [Fig. 2]. Not all treatment groups significantly decreased osteocyte survival compared to the non-treated culture control sample. The radiation-induced cell death groups (X-ray and UV light) showed no significant decrease in cell viability. Thirty minutes X-ray exposure declined the number of viable osteocytes per mm^2 ^bone matrix area from 119 (average of non-treatment control) to an average of 97 (+/- 46) surviving cells. Treatment with UV light demonstrated a similar cell survival decrease to 93 (+/- 44) average viable osteocytes per mm^2^. The number of surviving osteocytes was however significantly reduced by exposure to salt-free water (p ≤ 0.05), with an average of 45 (+/- 74) surviving osteocytes per bone matrix area [Fig. 2]. Moreover, in all of treatments - X-ray radiation, UV light exposure and salt-free water - a great standard deviation of cell survival was detected. The lowest number of surviving osteocytes after treatment, in combination with a low standard deviation, was detected after repeated freeze thawing cycles. An average of only 6 (+/- 6) surviving osteocytes per mm^2 ^bone area was detected after this treatment.

The positive treatment control, which experienced 56°C over night heat incubation, showed an average of 5 (+/- 10) viable osteocytes per bone matrix area. A significant difference in osteocyte survival after treatment with either radiation treatments (X-ray and UV light) to the positive treatment control was demonstrated. However, exposure to salt-free water or repeated freeze thawing cycles did show a decrease in osteocyte survival, which was not significantly different to the 56°C heat incubation control group.

## Discussion

Within this study ovine cancellous bone explants were successfully treated to induce osteocyte death. Moderate heat incubation is one of the most commonly used methods for the treatment of allogenic bone explants prior to transplantation. While the conventionally used sterilization methods with vapor temperatures over 100°C (121°C for 20 min, or 134°C for 5 min) lead to extreme loss of bone strength and osteoconductivity [6]. Different authors report that a temperature of 50°C is efficient to kill bone cells via thermal necrosis [7]. We chose to use an 18 h over night incubation at 56°C as positive treatment control, as it was previously shown to induce cell death in 5 × 10 mm cancellous bone explants [5]. However, this method is time-consuming and seems most impractical to be used as a cell-free control group.

The exposure of ovine cancellous bone explants to either UV-C or X-ray radiation showed no significant decrease in osteocyte survival over the 2 days culture control. Cell viability was significantly greater than the 56°C over night heat incubation control. X-rays are a form of electromagnetic radiation with a wavelength range of 10-0.1 nm. In medicine X-rays are mainly used for diagnostic imaging - radiography, or computer tomography of skeletal fractures [8]. Due to their high energy, X-rays are able to ionize (remove electrons) atoms, and destroy chemical compounds by the formation of radicals i.e. reactive oxygen species. Radiation induced DNA damage caused by X-ray exposure ranges from double-stand breaks, to ionization of the desoxyriboses [9]. If the changes in the genetic material of a cell are too dramatic - as it is intended during therapeutically radiotherapy of cancer treatment - cells can undergo apoptotic cell death [10]. UV radiation in its shorter wavelengths - below 200 nm - is also known permit ionization. Additionally, UV-C radiation (100-290 nm) can be the cause of thymine dimers in the DNA which than stops the DNA polymerase during replication. Cell death can be induced [10]. For this purpose, UV-C radiation is commonly as sterilization method.

The exposure of cells to hypotonic solutions, such as salt-free water which was used within this study, is causing cell swelling that terminates in cell death. This occurrence has been used occasionally in clinics, where surgeons washed the abdominal cavity with distilled water with the intention of lysing isolated cancer cells which are left after surgery [11]. In an *in vitro *study Selzner *et al*. could show that human colon cancer cells undergo apoptotic cell death triggered by short-term exposure (1-5 min) to a hypotonic solution [12]. The amount of surviving osteocytes after salt-free water treatment was significantly smaller than the non-treated 2 days cultured control and non-significantly different from the 56°C over night heat incubated control group. However, within this group a high standard deviation of surviving osteocytes was determined, resulting in a relatively questionable prediction of a uniform overall cell death. Probably this result was due to the presence of remaining salts within the bone marrow, protecting the cells from the osmotic shock.

The most promising treatment for the generation of an acellular bone explant control to be used within an operating theatre is to our knowledge the repeated use of freeze-thawing cycles. This method is one of the classical procedures for experimental *in vitro *cell lysis [13]. The rapid freezing procedure causes cells to swell and ultimately break due to massive ice-crystal formation, which is normally avoided using cryo-preservatives and controlled 1°C/min freezing during cryo preservation of cells [14]. The method could be time consuming if thawing would be permitted at room temperature. Therefore we recommend a 5 min thawing period at 56°C to be performed.

We did not perform additional tests to evaluate the mechanical properties of the cancellous bone biopsies after treatment. From previous studies within our group, we know that the Young's modulus of cancellous bone pieces does not significantly alter due to over night treatment at 56°C (unpublished observations). The mechanical properties of cancellous bone cylinders (5 mm × 10 mm) taken from bovine humeri were investigated in a study by Borchers *et al*. (1995) after long-term, repeated freezing thawing cycles and compared to 'non-treated' control cylinders [15]. No significant differences in compressive strength, elastic modulus, or mineral density could be determined. Therefore, we are likely to expect that the material properties of the rapid freeze-thaw treated bone explants in our study were not affected by treatment.

## Conclusion

Taken together, we demonstrate that a rapid cycle of freeze-thawing is an efficient, reliable, quick and easy method for the generation of acellular bone explants to be used as controls in bone graft studies.

## Competing interests

The authors declare that they have no competing interests.

## Authors' contributions

KJ performed most of the practical work, planned the experiments, analyzed the data and prepared the manuscript. VB participated in the preparation of the bone cores and exposure of the samples to the different treatments, performed the microscopic analysis and helped with the planning and preparation of the manuscript. PIF and AES participated in the cutting and staining of the bone tissue. RGR participated in the planning of the experiments, data analysis and the preparation of the manuscript. MJS supervised the study planning, data analysis and preparation of the manuscript. All authors read and approved the final manuscript.
